# Prediction Equations for In Vitro Ileal Disappearance of Dry Matter and Crude Protein Based on Chemical Composition in Dog Diets

**DOI:** 10.3390/ani13121937

**Published:** 2023-06-09

**Authors:** Yoon Soo Song, Beob Gyun Kim

**Affiliations:** Department of Animal Science, Konkuk University, Seoul 05029, Republic of Korea; sang8sys@konkuk.ac.kr

**Keywords:** dogs, dry matter, fiber, in vitro ileal disappearance, prediction equations, protein

## Abstract

**Simple Summary:**

Protein digestibility is important because protein consists of amino acids that are essential to dogs. As various feed ingredients, including plant or animal ingredients, are used in dog diets, protein digestibility values are variable among diets due to different diet formulations and nutrient compositions in the ingredients. In addition, protein digestibility may also be affected by feed processing procedures. However, animal experiments using dogs are expensive and time-consuming, and it has become a great concern for animal welfare and ethical issues. In this study, the nutrient digestibility of 18 commercial dog diets was measured using in vitro assays and prediction equations were developed for estimating in vitro nutrient disappearance of dog diets. The nutrient digestibility of dog diets was negatively correlated with fiber contents. Equations for estimating ileal protein digestibility of dog diets were developed: digestibility of crude protein (%) = 0.33 × crude protein (% as-is) − 0.49 × neutral detergent fiber (% as-is) + 81.25. Overall, in vitro ileal nutrient disappearance in a commercial dog diet can be estimated using the equations with protein and fiber.

**Abstract:**

The aims of this study were to determine in vitro ileal disappearance (IVID) of dry matter (DM) and crude protein (CP) in commercial dog diets and to develop equations for predicting the IVID of DM and CP in dog diets based on chemical composition. Eighteen commercial dog diets were analyzed for IVID of DM and CP using a two-step in vitro procedure for dogs. The diet samples in flasks with digestive enzymes were incubated for 2 h and 4 h to simulate digestion in the stomach and the small intestine, respectively. The contents of CP, ether extract, neutral detergent fiber (NDF), and ash in the diets ranged from 14.4 to 42.5%, 3.5 to 23.5%, 6.4 to 34.6%, and 4.9 to 10.0%, respectively, on an as-is basis. The NDF contents were negatively correlated with the IVID of DM and CP (r = −0.73 and r = −0.62, respectively; *p* < 0.05). The most suitable prediction equations for the IVID of DM and CP in the dog diets were: IVID of DM (%) = 81.33 + 0.46 × CP − 0.77 × NDF, R^2^ = 0.78; IVID of CP (%) = 81.25 + 0.33 × CP − 0.49 × NDF, R^2^ = 0.64, where all nutrients were in % on an as-is basis. In conclusion, dry matter and protein utilization of dog diets based on in vitro digestibility assays can be estimated fairly well using protein and fiber concentrations as independent variables.

## 1. Introduction

As the pet food industry grows rapidly, the demand for high-quality pet food increases with concerns from pet owners about the health of their pets [1,2]. Nutrient digestibility is an important indicator for evaluating the quality of ingredients and diets, and an accurate determination of nutrient digestibility can contribute to animal health and welfare [1,3]. Protein digestibility is particularly important because protein consists of amino acids that are essential to dogs. However, sufficient information on the protein digestibility of the ingredients used in dog diets is lacking. The European Pet Food Industry Federation suggested the minimum recommendation for dietary protein concentrations with an assumption of apparent protein digestibility of 80% [4]. As various feed ingredients, including plant- or animal-origin ingredients, are used for dog diets, protein digestibility values are variable among dog diets due to different diet formulations and nutrient compositions [3,5]. In addition, protein digestibility may also be affected by processing procedures for ingredients and dog diets due to the changes mainly in protein and fiber [6,7] or the Maillard reactions between amino acids and sugars [8,9]. Therefore, an accurate determination of protein digestibility in an individual diet is critical for dogs.

Animal experiments are required for an accurate determination of protein digestibility in dogs. Ileal-cannulated dogs have been used to remove the potential influence of microbial fermentation in the hindgut [2,10]. However, animal experiments using ileal-cannulated dogs are expensive, laborious, and time-consuming [7], and it has become a great concern about animal welfare and ethical problems, particularly in Europe in which using companion animals for experiments has been regulated strictly by the EU [11]. Although cecectomized rooster assays are alternatively used to determine the nutrient digestibility of pet diets [12,13], these assays require cecectomization procedures and animal experiments. On the other hand, in vitro assays are inexpensive and time-saving compared with animal experiments and can be used to evaluate the utilization of nutrients in diets under a laboratorial environment [14] based on a high correlation with the digestibility values obtained from animal experiments [15,16].

The in vitro disappearance of nutrients in commercial dog diets has been tested in some previous studies [1,15,17]. Although considered more convenient to conduct than in vivo assays, in vitro assays are also laborious and costly. Prediction equations based on chemical composition in dog diets would be one of the alternative methods for evaluating protein utilization in dog diets. However, the data for the correlation between chemical compositions and in vitro disappearance in commercial dog diets are limited, and, to our knowledge, the prediction equations based on chemical composition for in vitro disappearance of commercial dog diets have not been documented. The objectives of this study, therefore, were to determine the in vitro ileal disappearance (IVID) of dry matter (DM) and crude protein (CP) in commercial dog diets based on a two-step in vitro assay and to develop the equations for predicting IVID of DM and CP using chemical composition in dog diets as independent variables.

## 2. Materials and Methods

### 2.1. Commercial Dog Diets

Eighteen commercial dry extruded dog diets were used to determine the IVID of DM and CP (Table 1). The 18 diets were selected to cover a wide range of CP and fiber concentrations. Crude protein concentrations in the 18 diets ranged from 14.4 to 42.5%, ether extract (EE) ranged from 3.5 to 23.5%, neutral detergent fiber (NDF) ranged from 6.4 to 34.6%, and ash ranged from 4.9 to 10.0%.

### 2.2. Two-Step In Vitro Procedures

A two-step in vitro assay was performed to measure IVID of DM and CP in the diets by simulating the digestion processes in the stomach and the small intestine of dogs [15]. Briefly, the diets were finely ground (<1.0 mm) and weighed 1 ± 0.001 g. In the first step, 1 g of a diet sample was transferred into a 100 mL conical flask and then 25 mL of sodium phosphate-buffered solution (0.1 M, pH 6.0) and 10 mL of HCl (0.2 M, pH 0.7) were added to the flask. To simulate digestion conditions in the stomach of dogs, the pH was adjusted to 2.0 using 1 M HCl or 1 M NaOH solution, and 1 mL of freshly prepared pepsin solution (10 mg/mL; ≥250 units/mg solid, P7000, pepsin from porcine gastric mucosa; Sigma-Aldrich, St. Louis, MO, USA) was added to the flask. To avoid bacterial fermentation, 1 mL of chloramphenicol (C0378, chloramphenicol; Sigma-Aldrich, St. Louis, MO, USA) solution (5 g/L ethanol) was also added. The flasks were closed with a silicon stopper and incubated in a shaking incubator (LSI-3016R; Daihan Labtech, Namyangju, Republic of Korea) at 39 °C and 125 rpm for 2 h.

After the incubation, the second step mimicked the digestion and absorption in the small intestine of dogs. Firstly, 10 mL of phosphate-buffered solution (0.2 M, pH 6.8) and 5 mL of 0.6 M NaOH solution were added to the flasks. Then, the pH was adjusted to 6.8 using 1 M HCl or 1 M NaOH solution, and 1 mL of freshly prepared pancreatin solution (100 mg/mL; 4 × USP, P1750, pancreatin from porcine pancreas; Sigma-Aldrich, St. Louis, MO, USA) was added to the flasks. Thereafter, the flasks were incubated in the shaking incubator (LSI-3016R; Daihan Labtech, Namyangju, Republic of Korea) at 39 °C and 125 rpm for 4 h. After the incubation, 5 mL of 20% sulfosalicylic acid solution was added, and the flasks were left at room temperature for 30 min to precipitate the indigestible protein. After 30 min of precipitation, undigested samples were filtered through pre-dried and pre-weighed glass filter crucibles (Filter Crucibles CFE Por. 2; Robu, Hattert, Germany) containing 500 mg of acid-insoluble ash (Celite 545, Daejung Co., Ltd., Gyeonggi-do, Republic of Korea), which was used to inhibit plugging the filter by the potentially gelatinous residues. The flasks were rinsed twice with 1% sulfosalicylic acid solution, and 10 mL of 95% ethanol and 10 mL of 99.5% acetone were added twice to the glass filter crucibles. The filter crucibles with undigested residues were dried at 80 °C for 24 h. After cooling in a desiccator for 1 h, the filter crucibles were weighed to calculate the IVID of DM in the dog diets. The undigested residues in filter crucibles were collected and analyzed for CP contents to calculate IVID of CP. During the two-step in vitro procedure, a blank flask was included to correct the DM and CP contents in the residues that were not originated from the dog diets. The in vitro assay was performed in triplicate for each dog’s diet.

### 2.3. Chemical Analyses

All diets used in this study were finely ground (<1.0 mm) for chemical analyses. Diet samples were analyzed for DM (method 930.15; AOAC, 2019), CP (method 990.03; AOAC, 2019), EE (method 920.39), and ash (method 942.05; AOAC, 2019). Neutral detergent fiber (method 2002.04; AOAC, 2019) concentrations in diet samples were analyzed with a heat-stable amylase and expressed inclusive of residual ash. Undigested residues after the in vitro assay were analyzed for DM and CP as described in AOAC [18]. All chemical analyses were performed in duplicate.

### 2.4. Calculations

The IVID of DM was calculated using the following equation [14]:

IVID of DM (%) = [DM_diet_ − (DM_residue_ − DM_blank_)]/DM_diet_ × 100

where DM_diet_ (g) is the amount of diet on a DM basis, DM_residue_ (g) is the amount of residue on a DM basis after in vitro digestion procedures, and DM_blank_ (g) is the amount of residue on a DM basis after in vitro digestion procedures in the blank. The IVID of CP was calculated using the following equation [14]:

IVID of CP (%) = [(DM_diet_ × CP_diet_) − (DM_residue_ × CP_residue_) + (DM_blank_ × CP_blank_)]/(DM_diet_ × CP_diet_) × 100

where CP_diet_, CP_residue_, and CP_blank_ are the CP concentrations (%) expressed as DM basis in the diet, the undigested residue, and the blank, respectively.

### 2.5. Statistical Analyses

Data of nutrient compositions and IVID of DM and CP were analyzed for normal contribution using the UNIVARIATE procedure of the SAS (SAS Inst. Inc., Cary, NC, USA). The model included dog diet as a fixed variable. Correlation coefficients among the chemical composition (CP, EE, NDF, and ash) and IVID of DM and CP in dog diets were determined using the CORR procedure of SAS. Prediction equations for IVID of DM and CP were developed via the REG procedure using CP, EE, NDF, and ash in diets as independent variables. A flask was considered an experimental unit. The statistical significance was determined as *p* < 0.05.

## 3. Results

The IVID of DM in 18 commercial dog diets ranged from 69.7 to 88.1%, and the IVID of CP ranged from 72.2 to 88.7% (Table 2). The IVID of DM and IVID of CP had coefficient of variation values of 7.6 and 5.2%, respectively.

The NDF (r = −0.73, *p* < 0.001; Table 3) and ash (r = −0.51, *p* < 0.05) were negatively correlated with IVID of DM. The NDF (r = −0.62, *p* < 0.01) and ash (r = −0.47, *p* < 0.05) were negatively correlated with IVID of CP. The best-fitting model for IVID of DM and CP in commercial dog diets were as follows: IVID of DM (%) = 81.33 + CP × 0.46 − NDF × 0.77 with R^2^ = 0.78 and *p* < 0.001; IVID of CP (%) = 81.25 + CP × 0.33 − NDF × 0.49 with R^2^ = 0.64 and *p* < 0.001 (Table 4). All chemical compositions of diets are expressed as % as-is.

## 4. Discussion

Various feed ingredients are used for formulating dog diets at different inclusion rates depending on the breed and age of dogs to meet their nutrient requirement estimates [19] and the functional purposes, including gut health improvement [20] and obesity prevention [21]. Animal-origin ingredients commonly used in dog diets include fish meal, meat and bone meal, and chicken meat, which affect the concentrations of protein and ash in diets [14,22,23]. In contrast, fiber concentrations in diets are mainly affected by the inclusion rates of plant-origin ingredients [23,24,25]. For these reasons, nutrient compositions largely vary among dog diets. Due to the different ingredient and nutrient compositions, nutrient digestibility may also differ among dog diets. As the quantity of nutrients required by a dog is a digestible value rather than an intake value, an accurate determination of nutrient digestibility is critical in dog diets. However, using dogs as experimental animals for the evaluation of nutrient utilization is considered unethical and has been regulated in multiple countries [1,11]. The first objective of this work was, therefore, to determine the IVID of DM and CP in commercial dog diets using a two-step in vitro assay. The second objective was to develop equations for predicting the IVID of DM and CP in dog diets fed to dogs, which has not been previously documented to the best of our knowledge.

In vitro assays can be used for estimating the nutrient availability of non-ruminants [15,17,26,27]. In this study, a two-step in vitro procedure for dogs was adopted, which was modified from the procedure used for pigs by lowering the doses of exogenous digestive enzymes considering the shorter gastrointestinal tract and higher passage rate of digestion in dogs compared with pigs [15]. Previously, the linear relationship between in vivo CP digestibility and in vitro CP digestibility has been reported in dog diets by Hervera, Baucells, González, Pérez, and Castrillo [15].

The dog diets used in this study had similar ranges of EE and ash, but a wider range of CP contents compared with previous studies [1,15,28] in which in vitro assays were conducted to determine the utilization of nutrients. Although the range of NDF contents in this study cannot be compared directly with the range of crude fiber contents in previous studies, a fairly wide range of NDF contents (6.4 to 34.6%) was observed. The NDF concentrations are dependent upon the fiber concentrations in the ingredients and their inclusion rates. Plant-origin feed ingredients used in dog diets contain different fiber concentrations and perhaps have been included at different inclusion rates in the diets. When developing prediction equations, a wide range of independent variables enables the equations applicable to more diets [29,30,31].

The positive correlation between CP and NDF in the dog diets observed in this study is likely due to the dog diet formulator’s intention to maintain high or moderate fiber concentrations in high-CP diets [32,33,34]. When formulating high-CP diets, a large quantity of animal proteins is included, which results in low fiber concentrations unless additional fiber sources are used. Both fiber and protein have been suggested to be effective in reducing hunger and controlling the feed intake of dogs [32]. Thus, it is likely that a certain quantity of fibrous ingredients, such as beet pulp, fruit fibers, and sweet potato, as well as crystalline fibers, would have been included in the high-CP commercial diets used in this study although the inclusion rates of the ingredients are not disclosed. The positive correlation between CP and ash in the diets is likely due to the high ash contents in the animal protein sources such as fish meal, meat and bone meal, and chicken meat. The positive correlation between CP and EE is likely due to the addition of fats or oils in the high-CP diets manufactured for puppies to meet the high energy requirements [35,36]. Additionally, the relatively high contents of EE in animal protein sources may also have at least partially contributed to the correlation between CP and EE in dog diets.

The negative correlation between NDF contents and IVID of DM observed in this study indicates that nutrient utilization in dog diets decreases with increasing fiber contents. The present results are in agreement with those in previous studies which reported the negative effects of fiber on energy and nutrient digestibility in dogs [24,37,38,39]. Fibers are less digestible than starch, protein, and fat due to the lack of fiber-degrading enzymes secreted in the stomach and the small intestine of dogs, although microbes in the large intestine may partially digest dietary fiber [40,41,42]. In addition, fibers potentially disturb the degradation of other nutrients by exogenous enzymes [43], which explains the negative correlation between dietary NDF and IVID of CP in this work. Thus, the negative coefficient for NDF in the equation predicting IVID of DM and IVID of CP is reasonable. In other non-ruminant species, NDF has been used as an independent variable in the prediction equations for estimating ileal nutrient digestibility [44,45,46].

Although the CP content was not correlated with IVID of DM, the inclusion of CP as an independent variable along with NDF in the prediction equations resulted in improved determination coefficients (R^2^ = 0.78 vs. 0.53), indicating that the inclusion of CP in the model explains an additional 25 percentage units of the variability in the IVID of DM. The positive coefficient for CP in the model indicates that IVID of DM increases with increasing CP contents in the diet. In the correlation analysis, however, the IVID of DM was not correlated with CP, likely due to the fact that the magnitude of reduction in the IVID of DM by increasing NDF was substantially greater than the increase in the IVID of DM by increasing CP. With the same token, improving the accuracy of the equation (R^2^ = 0.64 vs. 0.39) for estimating the IVID of CP by including CP in the model with no correlation between the CP and IVID of CP can be explained by the influence of NDF and CP on IVID of CP.

The positive coefficient for CP in the equations for predicting IVID of DM and IVID of CP can be explained by the use of animal protein sources in high-CP dog diets. High-CP diets generally contain a large number of animal meats or animal protein hydrolysates that are highly digestible for dogs [2,47]. On the other hand, medium- and low-CP diets often contain a large number of plant ingredients and animal byproducts as protein sources that are less digestible for dogs. Some plant ingredients, including rapeseed meal and grain feeds, have been reported to be less digestible compared with animal proteins [48], whereas the CP digestibility of soybean meal has been reported to be comparable to that of poultry meal [49,50]. In addition, Murray et al. [51] reported lower digestibility of CP in meat and bone meal, one of the most used animal byproducts, compared with that in chicken meat.

To our knowledge, we first report the equations for estimating the ileal digestibility of dog diets based on nutrient compositions. The digestibility of DM and CP in commercial dog diets can be fairly accurately estimated by analyzing only protein and NDF. As the dog diets used in this study were selected to have wide ranges of CP and NDF contents, the equations for predicting IVID of DM and CP can be applicable to a wide range of commercial dog diets [29,30,31].

## 5. Conclusions

The nutrient digestibility of 18 commercial dog diets determined using a 2-step in vitro procedure had a large variability which was mainly due to the fiber and protein contents. In vitro ileal disappearance of dry matter and crude protein in a commercial dog diet can be estimated using the equations with protein and fiber.

## Figures and Tables

**Table 1:** Chemical composition of commercial dog diets, % as-is.

Diet	Age	Dry Matter	Crude Protein	Ether Extract	NDF	Ash
A	All life stages	92.1	14.4	4.7	20.0	7.1
B	Adult	90.8	14.8	5.2	9.6	6.1
C	All life stages	91.4	16.6	5.3	18.8	5.9
D	Young adult (>12 months)	91.3	18.5	6.5	19.9	9.6
E	Adult	91.4	20.3	10.4	6.4	6.3
F	All life stages	91.5	20.9	9.1	21.6	7.5
G	All life stages	92.2	21.2	3.5	17.6	6.2
H	All life stages	76.7	22.0	12.4	9.6	5.9
I	Adult	91.1	23.5	12.1	17.7	4.9
J	All life stages	93.0	26.1	14.0	21.3	9.3
K	Senior	91.6	27.6	13.3	7.1	5.8
L	Puppy (<12 months)	91.2	27.7	9.7	25.7	8.0
M	Late pregnancy-lactate	92.9	29.1	19.4	8.4	8.0
N	Puppy (<12 months)	92.6	34.8	18.8	25.8	8.2
O	Puppy (<12 months)	96.0	36.0	23.5	30.1	9.5
P	All life stages	93.9	36.6	15.8	32.2	8.7
Q	All life stages	92.9	40.8	15.7	34.3	9.1
R	All life stages	96.4	42.5	13.8	34.6	10.0
Mean		91.6	26.3	11.8	20.0	7.6
Minimum		76.7	14.4	3.5	6.4	4.9
Maximum		96.4	42.5	23.5	34.6	10.0
Standard deviation		4.4	8.8	5.6	9.2	1.6
CV, %		4.9	33.3	47.4	46.1	20.8

CV = coefficient of variable; NDF = neutral detergent fiber.

**Table 2:** In vitro ileal disappearance (%) of dry matter and crude protein in commercial dog diets ^1^.

Diet	Age	Dry Matter	Crude Protein
A	All life stages	70.9	72.2
B	Adult	79.0	78.3
C	All life stages	72.7	79.6
D	Young adult (>12 months)	75.4	77.3
E	Adult	88.1	87.1
F	All life stages	70.8	80.7
G	All life stages	80.8	79.7
H	All life stages	83.8	79.9
I	Adult	86.1	85.6
J	All life stages	78.0	77.9
K	Senior	85.7	85.3
L	Puppy (<12 months)	74.3	79.5
M	Late pregnancy-lactate	86.7	88.7
N	Puppy (<12 months)	78.7	79.2
O	Puppy (<12 months)	73.9	76.1
P	All life stages	75.3	78.3
Q	All life stages	69.7	77.1
R	All life stages	76.3	77.3
Mean		78.1	80.0
Minimum		69.7	72.2
Maximum		88.1	88.7
Standard deviation		5.9	4.2
CV, %		7.6	5.2

CV = coefficient of variable. ^1^ Each mean represents 3 observations.

**Table 3:** Correlation coefficients among chemical composition (% as-is) and in vitro ileal disappearance (IVID) of commercial dog diets.

Item	EE	NDF	Ash	IVID of DM	IVID of CP
CP	0.78 ***	0.69 **	0.63 **	−0.14	−0.06
EE	-	0.32	0.46	0.13	0.18
NDF		-	0.72 ***	−0.73 ***	−0.62 **
Ash			-	−0.51 *	−0.47 *
IVID of DM				-	0.81 ***

CP = crude protein; DM = dry matter; EE = ether extract; NDF = neutral detergent fiber. * *p* < 0.05, ** *p* < 0.01, and *** *p* < 0.001.

**Table 4:** Prediction equations for in vitro ileal dry matter and crude protein (CP) disappearance in dog diets.

Item	Regression Coefficient Parameter, % as-is			Statistical Parameter		
	Intercept	CP	NDF	RMSE	R^2^	*p*-Value
In vitro ileal dry matter disappearance, %						
Equation 1	87.45	-	−0.47	4.19	0.53	<0.001
Standard error	2.4	-	0.11	-	-	-
*p*-value	<0.001	-	<0.001	-	-	-
Equation 2	81.33	0.46	–0.77	2.99	0.78	<0.001
standard error	2.29	0.11	0.11	-	-	-
*p*-value	<0.001	0.001	<0.001	-	-	-
In vitro ileal CP disappearance, %						
Equation 3	85.59	-	−0.28	3.36	0.39	0.006
standard error	1.94	-	0.09	-	-	-
*p*-value	<0.001	-	0.006	-	-	-
Equation 4	81.25	0.33	−0.49	2.67	0.64	<0.001
standard error	2.04	0.10	0.10	-	-	-
*p*-value	<0.001	0.006	<0.001	-	-	-

CP = crude protein; DM = dry matter; EE = ether extract; NDF = neutral detergent fiber.

## Data Availability

The data presented in this current work are available.
